# Gene Expression Profiling of Mitochondrial Oxidative Phosphorylation (OXPHOS) Complex I in Friedreich Ataxia (FRDA) Patients

**DOI:** 10.1371/journal.pone.0094069

**Published:** 2014-04-04

**Authors:** Mohammad Hossein Salehi, Behnam Kamalidehghan, Massoud Houshmand, Goh Yong Meng, Majid Sadeghizadeh, Omid Aryani, Shahriar Nafissi

**Affiliations:** 1 Department of Molecular Genetics, Tarbiat Modares University, Tehran, Iran; 2 Department of Pharmacy, Faculty of Medicine, University of Malaya (UM), Kuala Lumpur, Malaysia; 3 Department of Medical Genetics, National Institute for Genetic Engineering and Biotechnology, Tehran, Iran; 4 Department of Medical Genetics, Special Medical Center, Tehran, Iran; 5 Department of Preclinical Sciences, Faculty of Veterinary Medicine, Universiti Putra Malaysia, Selangor, Malaysia; 6 Institute of Tropical Agriculture, Universiti Putra Malaysia, Selangor, Malaysia; 7 Department of Neurology, Tehran University of Medical Sciences, Shariati Hospital, Tehran, Iran; Ben-Gurion University of the Negev, Israel

## Abstract

Friedreich ataxia (FRDA) is the most frequent progressive autosomal recessive disorder associated with unstable expansion of GAA trinucleotide repeats in the first intron of the *FXN* gene, which encodes for the mitochondrial frataxin protein. The number of repeats correlates with disease severity, where impaired transcription of the *FXN* gene results in reduced expression of the frataxin protein. Gene expression studies provide insights into disease pathogenicity and identify potential biomarkers, an important goal of translational research in neurodegenerative diseases. Here, using real-time PCR (RT-PCR), the expression profiles of mitochondrial (mtDNA) and nuclear DNA (nDNA) genes that encode for the mitochondrial subunits of respiratory oxidative phosphorylation (OXPHOS) complex I in the blood panels of 21 FRDA patients and 24 healthy controls were investigated. Here, the expression pattern of mtDNA-encoded complex I subunits was distinctly different from the expression pattern of nDNA-encoded complex I subunits, where significant (*p*<0.05) down-regulation of the mitochondrial ND2, ND4L, and ND6 complex I genes, compared to controls, were observed. In addition, the expression pattern of one nDNA-encoded gene, NDUFA1, was significantly (*p*<0.05) down-regulated compared to control. These findings suggest, for the first time, that the regulation of complex I subunit expression in FRDA is complex, rather than merely being a reflection of global co-regulation, and may provide important clues toward novel therapeutic strategies for FRDA and mitochondrial complex I deficiency.

## Introduction

Friedreich ataxia (FRDA; OMIM# 229300) is the most common autosomal-recessively inherited ataxia that begins in childhood and leads to death in early adulthood. Patients exhibit neurodegeneration of the large sensory neurons and spinocerebellar tracts, along with variable systemic manifestations that include hypertrophic cardiomyopathy, scoliosis, and diabetes mellitus. FRDA results from the partial loss of frataxin (*FXN*; Entrez Gene ID 2395), a small nuclear-encoded 18-kDa protein targeted to the mitochondrial matrix [1].

The mitochondrion is supported by the 37 mitochondrial DNA (mtDNA)-encoded genes, which are transcribed in two polycistrones regulated by the heavy- and light-strand promoters [2]. Thirteen of these genes encode for protein subunits of the oxidative phosphorylation (OXPHOS) machinery, which are known to closely interact with nuclear DNA (nDNA)-encoded subunits within four of the five OXPHOS complexes (complexes I, III, IV and V). The mutation rate of the coding mtDNA is higher by an order of magnitude than that of most coding nDNA [3], [4].

Most patients with FRDA have a pathogenic expansion of a trinucleotide repeat (GAA) within the first intron of the *FXN* gene, which is located on chromosome locus 9q13 [1], [5], [6], that impairs the transcription of frataxin, resulting in a significant reduction in mRNA and protein levels [7]. Less than 5% of patients with FRDA are compound heterozygotes with the GAA repeat in one allele and a frataxin point mutation, including missense, nonsense, or intronic mutations, in the other allele [8]. FRDA affects 1 in every 50,000 people [9], in which among affected individuals, the Caucasian and South Asian populations are overrepresented. In the general population, the repeat length is 30, while in FRDA patients the repeat length ranges from 66 to 1700 [6], [10].

Iron-sulfur (Fe-S) clusters (ISC) are essential cofactors of proteins that perform important functions, such as electron transfer, catalysis, DNA repair, tRNA thiolation, ribosome biogenesis, and iron regulation [11]–[13]. Fe-S cluster biogenesis occurs in the mitochondria, and disruption of the process, caused by frataxin deficiency, is associated with human diseases such as FRDA and diabetes [6]. Loss of frataxin function, therefore, can result in ISC-containing protein deficiency, decreasing mitochondrial respiratory chain activity [14], accumulation of iron in affected organs [15], faulty iron handling and impaired ISC synthesis [14]. Thus, a decrease in frataxin may also increase reactive oxygen species (ROS) produced by an increase in bioavailable iron [16], [17], and the lack of iron detoxification [18]. Dysfunctional biosynthesis of mitochondrial iron-sulfur clusters and deficiency of ISC enzyme activity produces a defect in heme, which in turn causes a loss of cytochrome C. Electron transport activity impairment results in higher levels of ROS production [19], [20]. Defects in ISC assembly is the primary event in frataxin-deficient cells [21], while ROS production is a secondary event [22].

Clinically, FRDA is characterized by multiple symptoms, including progressive gait and limb ataxia, dysarthria, diabetes mellitus and hypertrophic cardiomyopathy [23]. There is intramitochondrial iron accumulation in the heart, liver, nervous system and spleen of FRDA patients, as well as reduction in mitochondrial DNA, the Fe-S cluster-containing subunits of the mitochondrial electron transport chain (complex I–III), and the enzyme aconitase [24], [25].

Complex I deficiency is the most common cause of respiratory chain dysfunction, accounting for 50% of all cases. Pathogenic mutations have been identified in nuclear genes that encode both complex I structural subunits and complex I assembly factors, and in all seven of the complex I mtDNA-encoded subunit genes [26]. Mitochondrial respiratory complex I deficiency and oxidative stress have been reported to occur in this disease, but mtDNA and nDNA that encode for the subunits of the chain complex could be considered a candidate modifier factor for FRDA disease [27].

The coordinated expression of known OXPHOS genes goes beyond a mitochondrial or even OXPHOS pattern of expression, to the level of individual complexes, in which the levels of mRNA are influenced by common promoter elements or a feedback mechanism from the assembled complexes [28]. Furthermore, It has been suggested that the regulation of expression of the OXPHOS complex I subunits in humans is complex and tends to undergo divergence of sub-clustered expression, rather than reflect global co-regulation [29].

In this study, the expression pattern profiles of NDUFA4, one of the nDNA-encoded subunits of complex IV, and 16 mitochondrial complex I subunits, including 7 mtDNA and 9 nDNA-encoded subunits, were analyzed in FRDA patients in order to determine whether the complex I genes were expressed through co-regulation or through sub-clustering.

## Materials and Methods

### Specimen Collection and Ethical Statement

Twenty-one FRDA patients, including 9 females and 12 males with a mean age of 17.8 years from 16 unrelated families, were diagnosed with clinical features that are summarized in Table 1. Blood samples from 21 FRDA patients and 24 random individuals of mixed ethnicities (as healthy controls) were obtained from the Special Medical Center, Tehran, Iran.

**Table 1:** Clinical characteristics of the 21 FRDA patients.

No. of Patients	Gender	Consanguineous marriages	History of family	Age of onset	Age of Diagnosis	Limb Ataxia	Lower-Limb Areflexia	Decreased Vibration Sense	Extensor Plantar Response	Axonal Neuropathy	Dysarthri	Eye Movement Abnormality*	Optic Atrophy	Food Deformity	Diabetes or Glucose Intolerance	GAA Repeat
FRDA 01	F	None	4	13	17	+	+	+	+	+	+	N	_	+	_	384
FRDA 02	M	First	1	11	13	+	+	+	+	+	+	N	_	+	_	329
FRDA 03	M	First	1	14	16	+	+	+	+	+	+	N	_	+	_	426
FRDA 04	M	None	7	12	25	+	+	+	+	_	+	DS	_	_	_	566
FRDA 05	M	None	7	17	21	+	+	+	+	+	+	DS	_	+	_	269
FRDA 06	M	None	7	7	19	+	+	_	+	_	+	N	_	_	_	890
FRDA 07	M	None	1	5	21	+	+	_	+	_	+	SWJ	_	_	_	947
FRDA 08	M	None	1	6	19	+	+	_	None	_	+	_	_	_	_	878
FRDA 09	F	First	None	12	16	+	+	_	+	_	+	SWJ	+	_	_	498
FRDA 10	M	None	1	23	29	+	+	_	+	+	+	DS	_	+	_	247
FRDA 11	M	None	1	15	19	+	+	_	None	_	+	_	_	_	_	584
FRDA 12	M	None	1	15	15	+	+	+	+	_	_	_	_	_	_	618
FRDA 13	F	First	None	6	25	+	+	+	+	+	+	N	_	+	_	908
FRDA 14	F	None	3	13	28	+	+	_	+	+	+	N	_	+	_	405
FRDA 15	F	None	1	2	4	+	+	+	+	+	+	N	_	+	_	981
FRDA 16	M	First	1	12	26	+	+	+	+	+	+	N	_	+	_	432
FRDA 17	F	None	None	3	8	+	+	_	+	_	+	DS	+	_	+	812
FRDA 18	F	First	2	13	16	+	+	+	+	+	+	DS	_	+	_	479
FRDA 19	F	First	2	9	10	+	+	+	+	+	+	N	_	+	_	554
FRDA 20	M	First	1	15	17	+	+	+	+	_	+	N	_	_	_	360
FRDA 21	F	None	1	8	10	+	+	+	+	_	+	SWJ	_	_	_	714

*N: Nystagmus; DS: Dysmetric Saccades; SWJ: Square Wave Jerks.

The exclusion criterion for the healthy control group was any history of cancer, metabolic diseases or mitochondrial DNA-related diseases that may affect mtDNA and nDNA. Written informed consent, including consent to participate in the study and consent to publish, was obtained from the patients, parents on behalf of children, and healthy controls for the present study in accordance with the Special Medical Center and Medical Ethics Committee (Approval No. FF-18-2007).

### RNA Extraction and cDNA Synthesis

RNA was extracted using the High Pure RNA Isolation Kit (Roche, Germany) and the products were transferred to −80°C for storage. Total cDNA was produced using the RevertAid First Strand cDNA Synthesis Kit (Fermentas, USA), according to the manufacturer's protocol. The products were transferred to −20°C for storage. The cDNA was used for subsequent real time-PCR amplification of the nDNA-encoded NDUFA1, NDUFA4, NDUFA5, NDUFA10, NDUFA12, NDUFB10, NDUFB11, NDUFS2, NDUFC2, NDUFV1 genes, and the mtDNA-encoded ND1, ND2, ND3, ND4, ND4L, ND5 and ND6 genes. Glyceraldehyde 3-phosphate dehydrogenase (GAPDH) and beta–actin (B-ACT) were used as the reference genes for gene expression analysis. The real time-PCR products were transferred to −20°C for storage.

### Real time-PCR Analysis

**Figure 1:**
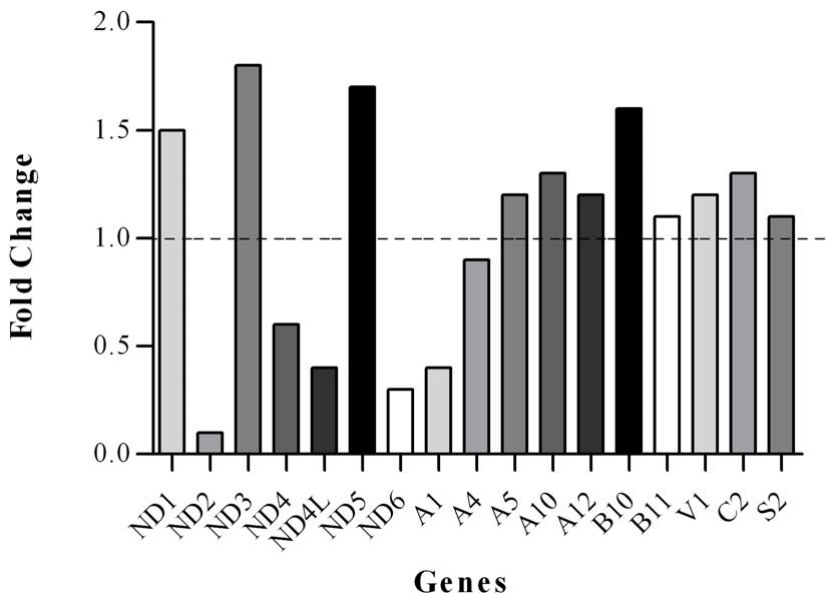
Gene expression pattern profiling of the nuclear and mitochondrial complex I genes as well as NDUFA4 from complex IV in FRDA patients compared to controls.

**Figure 2:**
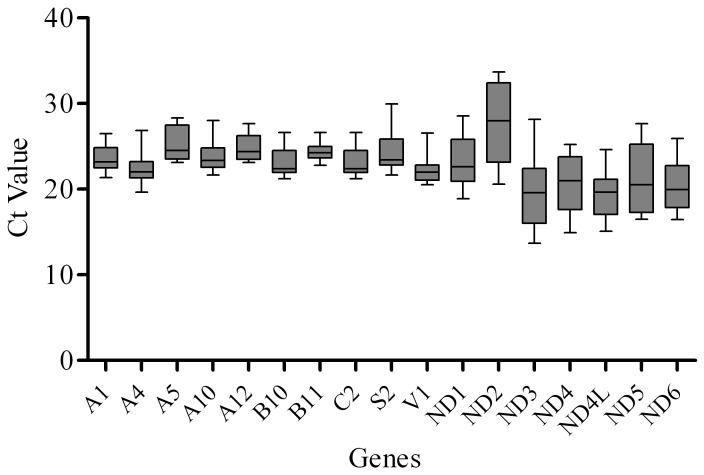
The mean Ct values of the nuclear and mitochondrial complex I genes as well as NDUFA4 from complex IV in FRDA patients compared to controls.

**Figure 3:**
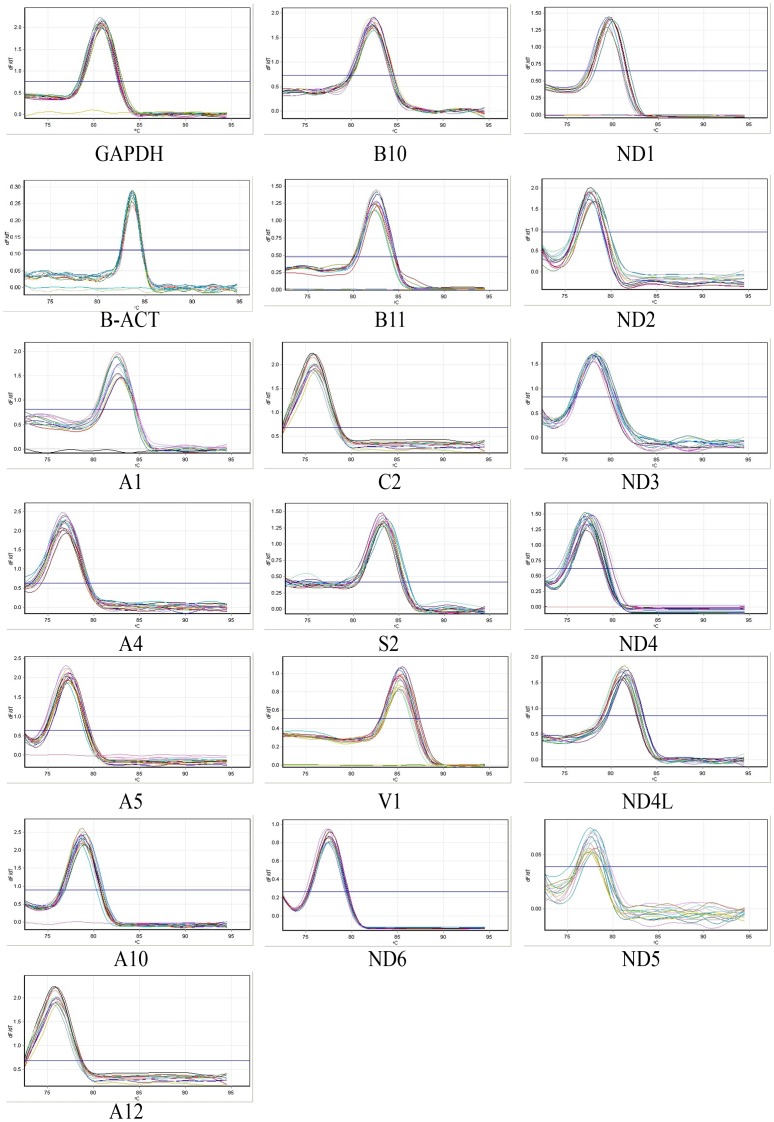
The melting curves of the nuclear and mitochondrial complex I genes as well as NDUFA4 from complex IV in FRDA patients compared to controls.

**Figure 4:**
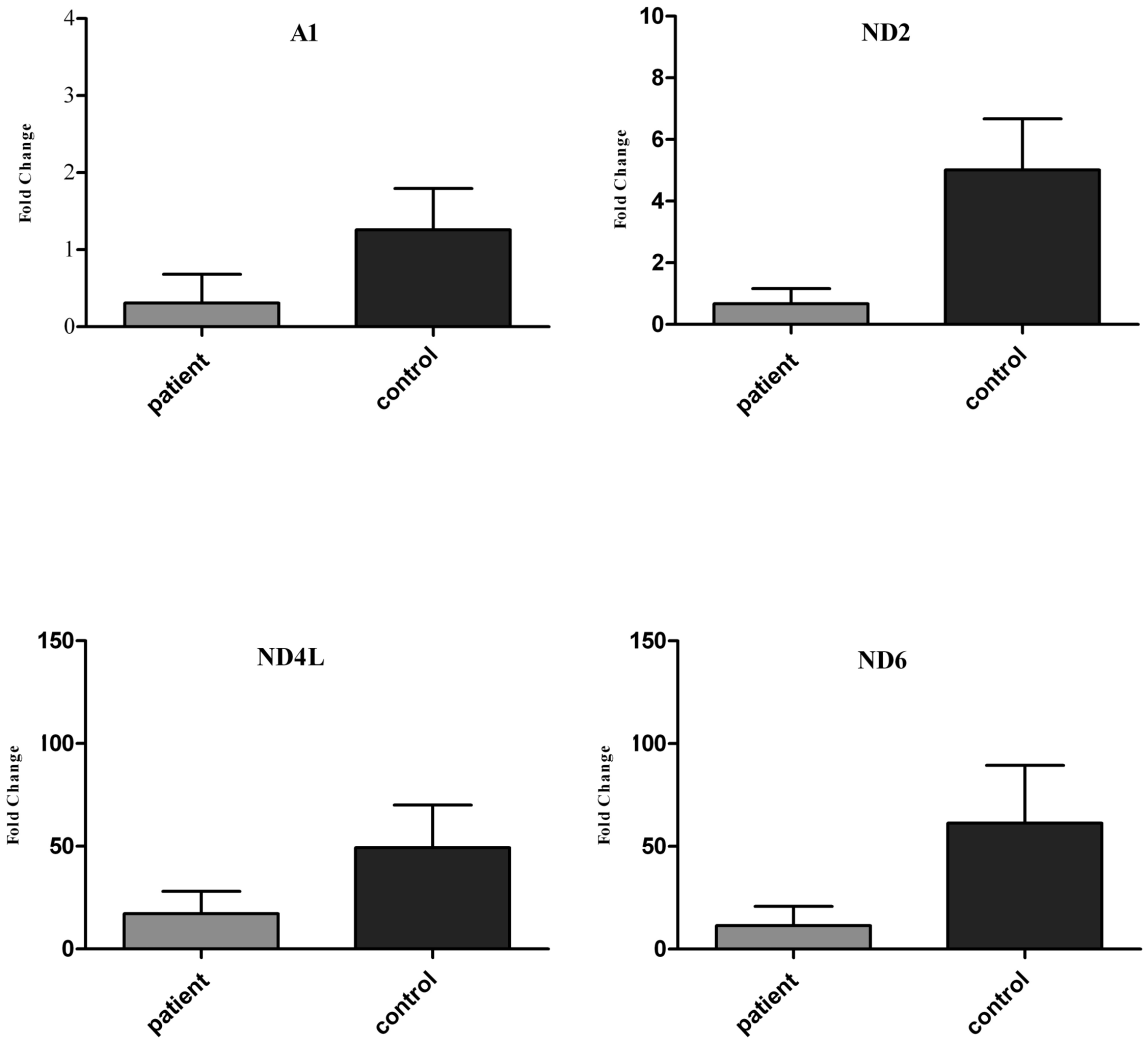
The nDNA and mtDNA gene expression profiles of complex I. The NDUFA1 nuclear gene and ND2, ND4L and ND6 mitochondrial genes of complex I were significantly (*p*<0.05) decreased in FRDA patients compared to controls.

Relative quantification of the transcript levels of the genes encoding for the nDNA and mtDNA subunits of complex I and NDUFA4, a subunit from complex IV, was investigated using real time-PCR (Table 2). 100–300 ng of cDNA from the 21 patients and 24 controls were subjected to real time-PCR amplifications in 20 μl reactions containing 1X Absolute SYBER Green ROX Mix, Thermo and 100 nM of each specific primer (Table 3). All primer pairs were initially tested via standard RT-PCR using the same conditions as described for real-time RT-PCR. Amplification of single products of expected size was verified by electrophoresis on 3% agarose gel and ethidium bromide staining (data not shown). The real time-PCR amplifications were carried out in a Rotor-Gene 6000 (Corbett) Real Time-PCR machine with the following protocol: 15 seconds at 95°C, followed by 40 cycles of denaturation for 30 seconds at 95°C, annealing for 1 minute at 60°C and extension for 30 seconds at 72°C. Each experiment was performed in duplicate tubes, and was repeated three times. To control for DNA contamination in the reaction mix, control tubes lacking DNA templates were included in duplicates, with the relevant set of primers in each experiment. Standard curves were generated in triplicates for each primer using ten-fold serial dilutions of a cDNA sample prepared from total RNA. PCR amplifications were also done without cDNA template, as negative control, to monitor the reagents utilized in the assay for possible contamination. The efficiencies of the eighteen pairs of primers were calculated. To determine their amplicon specificity, electrophoresis analysis of the real time-PCR products was also carried out. The real time-PCR Rotor-Gene 6000 software was used to determine the amplification cycle in which product accumulation was above the threshold (Ct). Real time-PCR Ct values (see Tables 4 and 5) were analyzed using the 2^−ΔΔCt^ method [30]. In each experiment, the mean Ct of duplicate tubes for a given gene was normalized to the mean Ct value of the reference gene (GAPDH). A1, A4, A5, A10, A12, B10, B11, S2, C2, V1 are abbreviations of the the nDNA-encoded NDUFA1, NDUFA4, NDUFA5, NDUFA10, NDUFA12, NDUFB10, NDUFB11, NDUFS2, NDUFC2, NDUFV1 genes, respectively. Additionally, the expression levels of the nuclear NDUFA5 and NDUFB10 genes and the mitochondrial ND1 and ND5 genes of complex I were investigated using B-ACT as a housekeeping gene (see Figures 1, 2, 3, and 4), in order to confirm the reliability of the results achieved with GAPDH as a housekeeping gene (Table 5).

**Table 2:** The tested complex I subunits, their genome affiliation (mtDNA or nuclear DNA), and their location in complex I/IV, as well as NDUFA4 from complex IV.

Complex I/IV	Gene Name	Locus (mtDNA/nDNA)	Location
Complex I			
	NDUFA1	nDNA	Hydrophobic arm
	NDUFA5	nDNA	Hydrophilic arm
	NDUFA10	nDNA	Hydrophobic arm
	NDUFA12	nDNA	Hydrophilic arm
	NDUFB10	nDNA	Hydrophobic arm
	NDUFB11	nDNA	Hydrophobic arm
	NDUFC2	nDNA	Hydrophobic arm
	NDUFS2	nDNA	Hydrophilic arm
	NDUFV1	nDNA	Hydrophilic arm
	ND1	mtDNA	Hydrophobic arm
	ND2	mtDNA	Hydrophobic arm
	ND3	mtDNA	Hydrophobic arm
	ND4	mtDNA	Hydrophobic arm
	ND4L	mtDNA	Hydrophobic arm
	ND5	mtDNA	Hydrophobic arm
	ND6	mtDNA	Hydrophobic arm
Complex IV			
	NDUFA4	nDNA	Hydrophobic arm

**Table 3:** Real time-PCR primers of selected nuclear and mitochondrial genes.

Gene Name	Primer Sequences	PCR Efficiency (%)	Tm (°C)	Amplicon Size (bp)
NDUFA1	F:ATGTGGTTCGAGATTCTCC R:GCAACCCTTTTTTCCTTGC	97.60	82.69	116
NDUFA4	F:CAGAGCCCTGGAACAAACTGGG R:GACCTTCATTCTAAAGCAGCG	96.69	76.82	137
NDUFA5	F:GAGAAGCTGGCTATGGTTAAAGCG R:CCACTAATGGCTCCCATAGTTTCC	96.18	77.18	154
NDUFA10	F: CACCTGCGATTACTGGTTCAG R:GCAGCTCTCTGAACTGATGTA	97.22	78.73	130
NDUFA12	F:ACATTCTGGGATGTGGATGG R:CTAGTGGTAGAATAAGGTAC	96.69	77.21	156
NDUFB10	F:TAGAGCGGCAGCACGCAAAG R:CTGACAGGCTTTGAGCCGATC	100.78	82.23	188
NDUFB11	F:GGAAAGCGGCCCCCAGAACCGAC R:CCACGCTCTTGGACACCCTGTGC	99.34	82.46	231
NDUFC2	F:GGTTTGCATCGCCAGCTTC R:CAGGAAAATCCTCTGGATG	102.47	75.87	137
NDUFS2	F:ACCCAAGCAAAGAAACAGCC R:AATGAGCTTCTCAGTGCCTC	97.22	83.30	214
NDUFV1	F:TGAGACGGTGCTGATGGACTTC R:AGGCGGGCGATGGCTTTC	99.34	85.22	113
ND1	3439H:CTACTACAACCCTTCGCTGAC 3655L:GGATTGAGTAAACGGCTAGGC	99.44	79.53	216
ND2	4892H:CATATACCAAATCTCTCCCTC 5166L:GTGCGAGATAGTAGTAGGGTC	90.08	77.85	274
ND3	10166H:TTACGAGTGCGGCTTCGACC 11455L:CCTAGTTTTAAGAGTACTGCG	90.64	78.19	189
ND4	11269H:CTAGGCTCACTAAACATTCTA 11455L:CCTAGTTTTAAGAGTACTGCG	96.74	77.23	186
ND4L	10528H:TAGTATATCGCTCACACCTC 10726L:GTAGTCTAGGCCATATGTG	96.63	81.33	198
ND5	3627H:TCGAATAATTCTTCTCACCC 13725L:TAGTAATGAGAAATCCTGCG	96.37	77.70	98
ND6	14359H:GTAGGATTGGTGCTGTGG 4258L:GGATCCTCCCGAATCAAC	97.98	77.75	119
GAPDH	F:GAAGGTGAAGGTCGGAGTC R:GAAGATGGTGATGGGATTTC	99.35	80.78	200
B-ACT	F:CGCGAGAAGATGACCCAGAT R:TCACCGGAGTCCATCACGAT	99.12	83.64	126

The PCR efficiency, melting temperature and amplicon size of the nuclear and mitochondrial complex I genes as well as NDUFA4 from complex IV.

**Table 4:** Summary of Real time-PCR results with GAPDH and B-ACT as reference genes.

Reference Gene	Gene Name	Ct Tg patient	Ct Tg Control	ΔCt TG	Ct HK patient	Ct HK Control	ΔCt HK	ΔΔCt	2^−(ΔΔCt)^
**GAPDH**									
	ND1	23.6	25.75	−2.15	21.53	23.11	−1.58	−0.57	1.5
	ND2	23.39	21.78	1.61	21.53	23.11	−1.58	3.19	0.1
	ND3	19.63	22.02	−2.39	21.53	23.11	−1.58	−0.81	1.8
	ND4	19.92	20.78	−0.86	21.53	23.11	−1.58	0.72	0.6
	ND4L	18.69	19.07	−0.38	21.53	23.11	−1.58	1.2	0.4
	ND5	21.37	23.75	−2.38	21.53	23.11	−1.58	−0.8	1.7
	ND6	19.61	19.35	0.26	21.53	23.11	−1.58	1.84	0.3
	NDUFA1	23.86	24.07	−0.21	21.53	23.11	−1.58	1.37	0.4
	NDUFA4	22.86	24.25	−1.39	21.53	23.11	−1.58	0.19	0.9
	NDUFA5	26.07	27.86	−1.79	21.53	23.11	−1.58	−0.21	1.2
	NDUFA10	24.55	26.48	−1.93	21.53	23.11	−1.58	−0.35	1.3
	NDUFA12	25.53	27.35	−1.82	21.53	23.11	−1.58	−0.24	1.2
	NDUFB10	23.63	25.85	−2.22	21.53	23.11	−1.58	−0.64	1.6
	NDUFB11	25.04	26.75	−1.71	21.53	23.11	−1.58	−0.13	1.1
	NDUFC2	23.6	25.58	−1.98	21.53	23.11	−1.58	−0.4	1.3
	NDUFS2	24.66	26.44	−1.78	21.53	23.11	−1.58	−0.2	1.1
	NDUFV1	23.86	25.75	−1.89	21.53	23.11	−1.58	−0.31	1.2
**B-ACT**									
	ND1	22.87	26.05	−3.18	22.36	23.85	−1.49	−1.69	3.2
	ND5	18.7	19.08	−0.38	22.36	23.85	−1.49	1.11	0.5
	A5	26.07	28.01	−1.94	22.36	23.85	−1.49	−0.45	1.4
	B10	24.27	26.38	−2.11	22.36	23.85	−1.49	−0.62	1.5

**Table 5:** Statistical analysis of the nuclear and mitochondrial complex I and IV genes.

Reference Gene	Genes	Patients/Controls	*p*- value
**GAPDH**			
	NDUFA1	21/24	0.0445*
	NDUFA4	21/24	0.1262
	NDUFA5	21/24	0.0566
	NDUFA10	21/24	0.2170
	NDUFA12	21/24	0.0622
	NDUFB10	21/24	0.0917
	NDUFB11	21/24	0.1878
	NDUFC2	21/24	0.1338
	NDUFS2	21/24	0.0717
	NDUFV1	21/24	0.1603
	ND1	21/24	0.1256
	ND2	21/24	0.0033*
	ND3	21/24	0.0674
	ND4	21/24	0.2075
	ND4L	21/24	0.0333*
	ND5	21/24	0.1330
	ND6	21/24	0.0037*
**B-ACT**			
	NDUFA5	21/24	0.4212
	NDUFB10	21/24	0.1056
	ND1	21/24	0.0941
	ND5	21/24	0.1597

The nDNA and mtDNA genes are normalized to GAPDH in FRDA patients compared to controls. The expression levels of four selected nDNA (NDUFA5 and NDUFB10) and mtDNA (ND1 and ND5) genes were normalized to B-ACT as a housekeeping gene in order to confirm the consistency of the result to those observed with GAPDH for quantitative gene expression analysis (*P*>0.05).

Statistical significance is expressed as *P<0.05 *vs* controls.

### Statistical analysis

Unpaired t-test analysis was used to examine the association between FRDA patients and healthy control groups, where *p*<0.05 is regarded as statistically significant. Statistical analysis was performed using Microsoft Office Excel 2010 and GraphPad Prism 6.0 software.

## Results

The presence of GAA repeat expansions on both alleles in 21 FRDA patients, including 12 males and 9 females, were confirmed using PCR. The gene expression of the nuclear and mitochondrial DNA genes encoding for complex I subunits were profiled using real time-PCR and the relative expression patterns of genes were normalized with GAPDH as the housekeeping gene. In order to evaluate amplification specificity and efficiency, electrophoresis was performed for the real time-PCR amplicons, where only a single band of the respective expected PCR product sizes were observed (data not shown).

Gene expression analysis was performed using the relative quantification method and compared to controls according to the Livak method [30] as summarized in Table 4. Additionally, the Ct range was assessed and the coefficient of variance for each gene across all samples was calculated in order to reveal the differences in transcript levels between the 17 nDNA and mtDNA genes.

In this study, the transcript levels of most mtDNA-encoded genes were lower than the transcript levels of the nDNA-encoded genes (Figure 1). However, the transcript variations of mtDNA-encoded genes were higher than that of the nDNA-encoded genes (Figure 2). The melting temperatures (Tm) of all PCR products ranged from 77.21°C for NDUFA12 to 85.22°C for NDUFV1 (Figure 3). The amplification efficiency of the primers varied from 90.08% for ND2 to 102.47% for NDUFC2. No primer dimers or non-specific amplification were observed. The PCR efficiency, melting temperature and amplicon size of selected genes are summarized in Table 3.

Our results indicated that there were significant differences at the transcript level between the nDNA and mtDNA subunit genes of complex I in patients compared to controls (Table 5). Specifically, mtDNA-encoded ND2, ND4L, and ND6 genes indicated significantly lower (*p*<0.05) gene expression than most of the other mtDNA-encoded genes in patients compared to controls, in which ND2 demonstrated the lowest relative gene expression level as compared to most of the mtDNA- and nDNA-encoded genes in patients compared to controls. Additionally, the relative gene expression level of the nDNA-encoded NDUFA1 subunit gene was lowest (*p*<0.05) compared to most of the nDNA-encoded genes in patients compared to controls and was expressed at a level similar to the mtDNA-encoded ND4L subunit gene in patients (Figure 4).

## Discussion

In this study, in order to identify the subset of key genes responsible for the clinical phenotype seen in FRDA patients, the gene expression profiles of OXPHOS complex I genes in patients were compared to the control groups. We hypothesized that mitochondrial and nuclear genes that encode for oxidative phosphorylation (OXPHOS) complex I subunits tend to undergo divergence and have sub-clustered expression rather than co-regulation. As previously reported, the transcription of many OXPHOS genes in type 2 diabetes mellitus, a metabolic disorder, is governed by co-regulation [31], [32], while another study revealed that the regulation of OXPHOS complex I subunit gene expression in humans is complex rather than reflecting global co-regulation [29]. Therefore, to examine gene expression as a co-regulation or sub-clustering mechanism, the 16 complex I subunit transcripts, including all seven mtDNA-encoded and nine nDNA-encoded subunits of complex I genes were investigated in FRDA patients. Additionally, one of the nDNA-encoded complex IV structural subunit genes, NDUFA4, formerly considered as a constituent of the complex I subunit, was also examined in this study [33].

Our findings showed the existence of two clusters of expression profiles in the OXPHOS complex I subunit genes, including all the mtDNA-encoded subunit genes and most of the tested nDNA-encoded subunit genes, where the mtDNA-encoded subunit genes are the major contributors to the divergence compared to the nDNA-encoded subunit genes, with co-regulated expression.

FRDA is caused by a GAA trinucleotide expansion in the first intron of the frataxin gene [10], leading to reduced expression of the mitochondrial protein frataxin. Frataxin is critically involved in iron sulfur cluster assembly, iron homeostasis [1], [8], and is a controller of intracellular oxidative stress by modulating the concentration of ROS [34]. In agreement with previous studies [1], [35], [36], our investigation revealed homozygous GAA triplet repeat expansions within the first intron of the *FXN* gene. Additionally, our results are clinically in line with previous studies [1], [6], [9], showing that FRDA is determined by progressive ataxia of gait and extremities, dysarthria and areflexia. Muscle weakness is evident particularly in the lower limbs, though signs of weakness or fatigue may be masked by more prominent features of FRDA.

It is noteworthy to mention that the frataxin is particularly abundant in tissues that are rich in mitochondria, such as liver, heart, pancreas, muscle, brown fat, thymus and kidney [6], [37], [38]. However, the choice of blood samples in the present study was due to scarce availability of the main target tissues. FRDA patients with low levels of frataxin may have heteroplasmic mtDNA mutations, where the combination of mitochondrial mutations, altered proteins, excess iron and increased ROS could contribute significantly to the disease state [39]. Deficiencies in the activities of the iron-sulfur cluster (ISC)-dependent aconitase, neural and cardiac cell degeneration as well as mitochondrial respiratory chain complexes I, II and III have been demonstrated in FRDA patients [40].

Accumulation and imbalance of iron can lead to oxidative stress, the generation of ROS and severe mitochondrial damage. Formation of ROS as a by-product of the OXPHOS process results in damaged DNA, RNA, proteins and lipids in many degenerative diseases, cancer and aging [39]. Frataxin deficiency causes more sensitivity to oxidative stress [35], mtDNA and nDNA instability, chromosomal mutations and enhanced recombination rates, probably through hydrogen peroxide intermediates [39].

Mitochondrial diseases with defects in the OXPHOS system are mediated by mutations in either nDNA or mtDNA, and therefore have distinct patterns of inheritance, depending on the genetic origin [41]. Complex I deficiency is the most common defect of the mitochondrial OXPHOS system, which accounts for about one third of all cases of OXPHOS disorders [42]. Analyses of complex I deficiency have revealed mutations in some nDNA-encoded and mtDNA subunit genes in OXPHOS disorders [41]. Our study showed the significant down-regulation of the nDNA-encoded subunit gene NDUFA1 and three mtDNA complex I genes ND2, ND4L and ND6 in FRDA patients compared to controls. A microarray-based analysis indicated evidence for co-transcription of OXPHOS genes, showing the preference of subunits of each OXPHOS complex to cluster separately [28]. It seems that mtDNA-encoded subunits are transcribed in a polycistronic fashion and are regulated by the same promoter (excluding ND6) [29], [43]. However, the variation of complex I gene expression response in our study, might be attributed to existence of post-transcriptional regulatory mechanisms and/or differences in mRNA stability among mtDNA-encoded genes. Additionally, post-transcriptional regulation was previously observed for mtDNA-encoded tRNA genes, suggesting that such regulation is not restricted to protein coding mtDNA genes [44].

## Conclusions

This study provides the first description of the gene expression patterns of 16 OXPHOS complex I subunit genes and the NDUFA4 gene, a subunit of complex IV - a total of 17 genes consisting of seven hallmark mtDNA-encoded genes and ten representative nDNA-encoded subunit genes in FRDA patients compared to controls. The expression of nDNA-encoded subunit genes clustered separately from those of the mtDNA-encoded subunit genes, in which most of the nDNA-encoded subunit genes, such as NDUFA4, NDUFA5, NDUFA10, NDUFA12, NDUFB10, NDUFB11, NDUFC2, NDUFS2, NDUFV1, revealed distinct co-regulated gene expressions, compared to the mtDNA-encoded subunit genes, indicating sub-clustering gene expression. Additionally, the down-regulation of the mtDNA-encoded ND2, ND4L and ND6 subunit genes revealed a divergence, due to mutations, from the rest of the mtDNA-endoded genes regulated by the same promoter. Taken together, our analysis on the mitochondrial complex I gene expression patterns has led to a better understanding of the therapeutic strategies and molecular mechanisms of FRDA in order to predict genetic risk and potentially pave the way for a new diagnostic tool for FRDA patients.
